# A Case of Phthisis Pulmonalis; with Remarks

**Published:** 1790

**Authors:** Edmund Pitts Gapper

**Affiliations:** Surgeon at Mere in Wiltshire


XIII. *A Cafe of Phthifis Pulmonalis; with Remarks. By Mr.* Edmund Pitts Gapper, *Surgeon at Mere in Wiltfhire.*

IN the beginning of July, 1789, I was defired to vifit, for the firft time, a young lady in a confirmed phthifis pulmonalis, and who a few days before had returned from Briftol in an almoft hopelefs ftate. She was about twentytwenty-three years of age, of a full habit, and lively; of a florid complexion and clear ſkin, accompanied with a ſomewhat enlarged thyroid gland, but no other diſtinguiſhing marks of ſcrophula.

I found her labouring under extreme debility, languor, and emaciation; a violent, troubleſome, and almoſt inceſſant cough, (which I was informed had been preceded by hæmoptyſis) attended with a copious expectoration of purulent matter; a hectic fever, with regular exacerbations; night ſweats, and a great tendency to diarrhœa. She had little ſleep, and experienced a total loſs of appetite and ſtrength; indeed the laſt was ſo far exhauſted, that ſhe was under the neceſſity of being carried to and from her bed, and was utterly incapable of ſtanding without ſupport. Her caſe appeared ſo truly deplorable and helpleſs, as would have juſtified any medical man in pronouncing her diſſolution to be at hand. Under theſe diſcouraging circumſtances I did not heſitate how to act. I conſidered that the inflammatory ſtage of her diſorder was paſt, and that it was the ſuppurative only which I had to combat. No marks of inflammation remained, no pains in the cheſt, no difficulty of breathing. The pulſe, pulfe, though as quick as 134 in a minute, yet was extremely low and weak, and the whole fyftem was in a ftate of debility. Thefe circumftances appeared to call loudly for a tonic plan of treatment, and induced me to adopt it. I was farther confirmed in thefe fentiments by having frefh in my recollection a cafe in the London Medical Journal, related by Dr. May, of a lady in a confirmed phthifis pulmonalis, who had recovered under a fimilar mode of treatment. His arguments on the fubject appeared to me to be plaufible and convincing, and I was ftruck with the rationality and juftice of them. I was, therefore, determined to make a fair trial of his method; and having explained my ideas on the fubject to her friends, obtained their confent to proceed as I thought beft. Her faline draughts and emulfions were accordingly thrown afide, the antiphlogiftic plan, which had hitherto been purfued, was abandoned, and draughts compounded of a ftrong decoction of red bark, aromatic confection, and compound tincture of lavender, were fubftituted. A little cinnamon and gum arabic were added to the decoction, to obviate any tendency to a diarrhœa, which, in her then reduced ftate, muft inevitably have proved fatal.

A fuffi-

A fufficient quantity of tincture of opium was adminiftered every night to procure fleep, and was increafed by degrees, without the leaft inconvenience, to feventy drops at each dofe. I alfo recommended it to her to make ufe of a more invigorating regimen than fhe had hitherto done. I ftrictly enjoined her to take frequently, nay every hour, a fmall quantity of fome nourifhing food, fuch as fago, falep, tipioca, jellies, chicken and mutton broths, &c. I allowed her light animal food for her dinner, and to drink three or four glaffes of wine in the courfe of the day, nay even in the night, at intervals, if fhe awoke. I ftrongly and earneftly recommended fwinging to her, but it was never complied with; a fedan chair was, however, procured, in which fhe was carried out, whenever the weather permitted, for a few minutes at each time, increafing it by degrees as her ftrength allowed. I alfo cautioned her not to lie late in bed, as being too relaxing, but rather to repofe at intervals during the day, if fhe felt fatigued.

This plan was fteadily and refolutely perfifted in for upwards of two months, and its effects I am now to relate. The pulfe, which, as I have faid, was at 134, was, in the fpace of a week or ten days, reduced to 100; in three weeks time to 90; and finally to near its natural ſtandard. As the frequency of the pulſe abated ſo did the fever, till it entirely left her. The expectoration grew gradually leſs in quantity, and at laſt conſiſted chiefly, but not entirely, of phlegm. She had comfortable nights; her appetite and ſtrength returned, and the latter in ſo eminent a degree as to enable her to walk frequently in the garden without any aſſiſtance, and to ride on horſeback ſingle twice every day.

Thus far every thing went well, and I entertained the moſt ſanguine hopes of her recovery; but ſhe at length refuſed to continue the bark any longer; and though an emetic removed the nauſea it occaſioned, yet it could not conquer her averſion to medicine, and it was totally abandoned; and neither the manifeſt benefit ſhe had received, nor the earneſt intreaties of her friends, could conquer this obſtinate reſolution. A fatal inſenſibility of the danger ſeemed to take poſſeſſion of her. Her medicines being thrown aſide, and her diet neglected, the former ſymptoms, which were nearly conquered, returned with aggravated force, and no means being ſuffered to be uſed to check them, them, they bore down all oppoſition, and in a ſhort time nature ſunk under them.

Though this caſe ended fatally, yet it undoubtedly contains facts which may be adduced as ſtrong and ſtriking proofs of the truth of Dr. May's obſervations; and I am firmly of opinion, that had the method which was entered on ſo vigorouſly and ſucceſsfully been duly perſevered in, that ſhe would have been at this moment a living inſtance of its efficacy. Indeed the ſuperior advantages of the tonic over the antiphlogiſtic plan in this caſe were ſo immediately and ſtrongly marked as muſt be obvious to the meaneſt capacity. It has been long lamented that all the art of medicine, the boaſted virtues of Briſtol water, with the ſalubrious air of a ſouthern climate, have ever failed. It is evident, then, we have been miſtaken in our ideas and treatment of this diſeaſe, and it follows that it is highly incumbent on us to adopt ſuch as reaſon and experience ſhall point out, It is aſtoniſhing that the faculty have ſo long perſiſted in a method which every day's experience convinced them was inefficacious; and if the fatal idea of a complaint being incurable is ſuffered to remain, there is then a total end of all hope of our ever attaining any degree of

 perfecperfection in this art of curing diseases. The cause of humanity strongly urges us to exert all our faculties to snatch from certain and inevitable death the vast number of our fellow creatures, whom we see every day languishing in consumptions, lost to all hope. That our ideas of the disease have been erroneous, is evident from the ill success of our treatment. A hectic, accompanying an expectoration of purulent matter, has usually been treated as inflammatory, without, it should seem, its having ever been considered that an inflammatory fever in all other instances, such as peripneumony, pleuritis, &c., terminates, in a fixed time, either by resolution, suppuration, or gangrene; for I deny that the fever which *follows* suppuration is of the same nature as that which *precedes* it. We often see, in large abscesses and carious joints, a true hectic produced from the absorption of pus, similar in every respect to that attending a confirmed phthisis pulmonalis; and is it not the practice to prescribe bark, opium, wine, and an invigorating regimen, in these cases? Will the same treatment, which is absolutely necessary, and which will effectually conquer a fever occasioned by the formation of a large abscess in a highly inflammatory state, I say will it cure a fever a fever produced by the abſorption of pus? Certainly not; and theſe arguments apply themſelves aptly and readily to the preſent ſubject. Phyſicians are agreed that a true hectic does not appear till pus is formed or expectorated; the fever is, therefore, the effect, not the cauſe. It is undoubtedly produced from the abſorption of pus, no matter from what part, whether from the lungs, large abſceſſes, or carious joints, and the ſame treatment ſeems to be as applicable to one as to the other. The long continuance and frequent recurrence of a hectic would lead to the rational concluſion, that it is not inflammatory; and I believe no one will deny that an inflammatory fever, with the pulſe at 130, attended with a violent cough and difficulty of breathing, ever fails of terminating, in the courſe of a few days, either in death or recovery. The extreme emaciation and debility, with the loſs of ſtrength and appetite, ſtrongly forbid the antiphlogiſtic plan, and loudly call for a more invigorating treatment, that is, when purulent matter is expectorated, and a hectic is the conſequence; for I imagine a different method is proper before either of theſe circumſtances have taken place, except when the diſeaſe is evidently ſcrophulous, for then a tonic plan feems to be indicated from the beginning. The cafe I have related is a ftrong proof that a hectic fever, produced by the abforption of pus, is not inflammatory, for in that cafe every fymptom would have been aggravated from the mode of treatment I perfued; whereas the direct contrary took place: the pulfe, inftead of being quickened, abated of its frequency; the expectoration fenfibly and materially decreafed; the ftrength and appetite returned, and every hope was awakened while the tonic plan was perfifted in; and the fatal alteration which took place on its being difcontinued is fufficiently convincing to confirm the moft incredulous.

To Dr. May every acknowledgement is due for having boldly, and in defiance of prejudice, adopted and recommended a plan evidently founded on reafon; and I think that every unprejudiced and impartial man muft allow, that as the general mode of treatment in this fatal difeafe has been hitherto almoft uniformly unfuccefsful, that it is high time fome other were adopted; and that, at the worft, it muft ever be far better to employ the *anceps remedium* of Celfus, than one on which, as long and repeated experience has proved, we cannot with with any confidence rely. It is to be hoped that medical practitioners will take every opportunity of enforcing this practice, and of communicating the refult to the world.

I fhall conclude thefe obfervations with a quotation from a treatife on this fubject by Dr. Ryan, replete with theoretical ingenuity and found reafoning.

"But when the body is become exhaufted "by the progrefs of the difeafe, when the "fluids are evacuated in confiderable quanti-"ties, when every mufcle and every fibre is "materially impaired in its functions, muft "the fame enervating practice be put in exe-"cution in every inftance of the difeafe? "Surely no authority, not even the prefump-"tion of experience, can warrant fo erroneous "a doctrine."

